# Rapid Resolution of Apophenic Psychosis With Low-Dose Olanzapine in Very Late-Onset Schizophrenia-Like Psychosis

**DOI:** 10.7759/cureus.116065

**Published:** 2026-09-10

**Authors:** Yusuf Jamal, Mohamed A Mohamed, Nauman Malik

**Affiliations:** 1 Psychiatry, University Hospital Galway, Galway, IRL; 2 Psychiatry, Letterkenny University Hospital, Letterkenny, IRL

**Keywords:** apophenia, delusional perception, geriatric psychiatry, late life psychosis, olanzapine

## Abstract

Very late‑onset schizophrenia‑like psychosis (VLOSLP) presents diagnostic complexity due to the broad range of medical, neurological, and psychiatric conditions that can manifest with late‑life psychosis. We describe a case of a man in his mid‑70s who developed progressive apophenic experiences, characterised by the perception of patterns, rhythms, and “algorithms” in everyday environments, leading to referential beliefs and behavioural change. Investigations showed normal blood tests, preserved cognitive screening scores, and MRI evidence of chronic white matter ischaemic changes and diffuse cerebral atrophy. Differential diagnoses included vascular psychosis, autoimmune encephalitis, obsessive-compulsive disorder with poor insight, and neurodegenerative disease; however, the overall presentation was most consistent with VLOSLP. The patient was treated using a bio‑psycho‑social approach, with limited response to quetiapine but rapid and sustained remission following initiation of low‑dose olanzapine. Symptom recurrence after dose reduction further supported a primary psychotic process. This case highlights an atypical presentation of VLOSLP dominated by apophenia and underscores the importance of thorough assessment and cautious, individualised antipsychotic treatment in older adults.

## Introduction

Late-onset psychosis refers to psychotic symptoms that emerge in older adulthood. It presents significant diagnostic challenges as psychotic symptoms in older adults arise from many different causes, such as medical, neurological, or psychiatric, several of which overlap in presentation. There is an increased prevalence of medical and neurological conditions that accompany advancing age, thereby making diagnosis more difficult [1]. Common secondary causes include neurodegenerative disorders and cerebrovascular disease, which may overlap with psychotic disorders. Late-onset psychosis encompasses a range of conditions, including late-onset schizophrenia, delusional disorder, psychosis associated with Parkinson's disease, delirium-related psychosis, and psychotic symptoms occurring alongside neurocognitive disorders such as dementias [2].

The classification of late-life psychotic illnesses has evolved significantly over time [3]. In 2000, experts established the term "Very Late-Onset Schizophrenia-Like Psychosis" (VLOSLP) [4]. This applies to patients whose initial psychotic symptoms emerge after the age of 60 [4]. Unlike early-onset schizophrenia, which is more prevalent in males, VLOSLP shows female predominance [5]. The condition is characterised mainly by positive psychotic symptoms [6]. Patients may experience multimodal hallucinations, which can be very distressing. The presence of persecutory delusions, which may include beliefs of being watched or harmed, is common [5]. Partition delusions are a hallmark, in which patients believe solid walls and floors can be penetrated [5]. This leads to the belief that others can pass through physical barriers to spy on or hurt them. Late-onset psychotic disorders such as VLOSLP are mostly associated with prominent persecutory and paranoid delusions, often accompanied by sensory impairments. These presentations are often fixed and difficult to treat. However, atypical presentations can occur. We report an unusual presentation of late-onset psychotic disorder in which the patient developed altered pattern perception and assigned meaning to environmental stimuli, consistent with apophenia.

## Case presentation

We present a case of a male in his mid-70s, previously working as a ward manager, who was referred by his general practitioner (GP) in November 2024 following an acute episode of behavioural disturbance while travelling in New York. During this episode, he became distracted by visual patterns in buildings and nearly stepped into traffic. Over the preceding five to six months, he had developed unusual experiences characterised by perceiving patterns, rhythms, and “algorithms” in everyday surroundings. These included interpreting traffic flow, television signals, animal behaviour, and environmental structures as interconnected systems shaped by recurring patterns or “algorithms” which he experienced as having special meaning. He believed these patterns carried personal significance and contained hidden messages relevant to him. He did not report seeing or hearing things that were not present. However, he assigned excessive significance to real environmental stimuli. There were associated referential beliefs, with behavioural impact such as altered walking patterns and repetitive actions to “break” perceived patterns. Collateral history indicated increasing preoccupation, mild persecutory ideas, and evening worsening of symptoms. There was no history of hallucinations, formal thought disorder, or significant mood disturbance. He remained independent in activities of daily living.

The patient's investigations demonstrated normal routine blood tests, with no metabolic or infectious abnormalities identified. An MRI brain imaging reported chronic white matter ischaemic changes alongside diffuse cerebral atrophy, consistent with small-vessel disease and age-related neurodegeneration. Cognitive assessment showed an ACE-III score of 93/100 and a Mini-ACE score of 26/30, indicating no significant evidence of a major neurocognitive disorder. An autoimmune encephalitis workup was requested to exclude inflammatory aetiologies, and it came back normal. ECG and metabolic monitoring were completed, with no acute concerns arising during physical health screening.

Differential diagnosis

Assessing new-onset psychosis in older adults requires a highly systematic clinical investigation. Any new-onset psychosis in a patient over 60 must be considered "organic until proven otherwise" [7]. A diagnosis of VLOSLP requires the systematic exclusion of secondary causes [7]. The most likely diagnosis in this case was very late-onset schizophrenia-like psychosis. The patient's age of onset, absence of previous psychiatric history, and the manifestation of positive psychotic symptoms make this a reasonable diagnosis [3]. Despite the fact that VLOSLP is usually associated with persecutory delusions and hallucinations, clinical presentations may include misinterpretation of environmental stimuli [3]. The patient's fixed beliefs regarding environmental “patterns” and “algorithms,” alongside behavioural changes driven by these interpretations, are consistent with a late-onset schizophrenia psychosis.

Microvascular brain lesions can disrupt frontal-subcortical circuitry [8]. This disruption can cause a vascular psychosis that mimics primary schizophrenia [8]. The patient's magnetic resonance imaging demonstrated chronic white matter ischaemic changes and diffuse cerebral atrophy. This raised the differential of vascular disease [8]. However, the patient demonstrated relatively preserved screening cognitive scores and maintained independence in activities of daily living. The patient scored a 93/100 on Addenbrooke's Cognitive Examination III (ACE-III). The ACE-III included assessment of executive functioning, including attention, planning, organisation and verbal fluency. This made the differential diagnosis of vascular aetiology less likely. The absence of marked impairment in these domains was relevant given that vascular pathology commonly affects executive ability. In this case, a score of 93/100 made the differential diagnosis of vascular aetiology less likely.

Autoimmune encephalitis was included as a differential due to the manifestation of behavioural and psychiatric symptoms in later life. The geriatric demographic is highly susceptible to autoimmune encephalitis [9]. Antibodies targeting LGI1 and CASPR2 frequently masquerade as primary late-life psychosis [9]. In this case, the absence of seizures, catatonia, and rapid neurological deterioration made this diagnosis less likely. A comprehensive autoimmune workup alongside other blood tests returned normal, providing further evidence against this diagnosis. Given the clinical information, this differential was considered less likely, although it cannot be completely excluded.

Obsessive-compulsive disorder (OCD) with poor insight was also considered, as the patient described repetitive behaviours and a need to correct or break perceived patterns in his environment. However, these behaviours were driven by fixed beliefs and there was little evidence of the anxiety typically seen in OCD. Although insight can vary in OCD, the International Classification of Diseases, 11th Revision (ICD-11) recognises that individuals with OCD may have absent or limited insight into the excessive or unreasonable nature of their obsessions and compulsions; however, persistent, fixed beliefs without associated obsessive thoughts or compulsive anxiety may be more suggestive of psychosis [10]. Overall, the presentation was most consistent with VLOSLP, characterised by prominent apophenic delusional phenomena in the context of preserved cognitive function and underlying structural brain changes.

Management of the condition

Management followed a bio-psycho-social approach, combining pharmacological treatment with structured psychosocial support. A carefully monitored trial of a low-dose atypical antipsychotic was considered. Within the UK and Ireland, quetiapine is often utilised as a default first-line agent [11]. It is especially useful if there is concern about extrapyramidal symptoms and frailty. In older adults, due to the increased risk of adverse effects, antipsychotics should be prescribed cautiously, and a low dose over a short period should be used initially [12]. The patient was commenced on quetiapine, which was gradually titrated; however, due to limited clinical response, a cross-taper to olanzapine 5 mg at night was undertaken. 

Olanzapine was found to be effective in this case, leading to a rapid and marked improvement, with full resolution of psychotic symptoms and restoration of insight into the unreality of his experiences. Psychosocial interventions included regular input from the psychiatric specialist nurse, anxiety-management strategies, distraction techniques, and engagement in goal-directed community activities to support functional recovery. Driving safety advice was provided, and the patient was followed closely in the community. He achieved full remission on olanzapine and returned to his baseline level of functioning. Follow-up to January 2026 confirmed sustained remission. At the patient’s request, the dose was reduced to 2.5 mg at night; however, early symptom recurrence prompted a return to olanzapine 5 mg, after which he again remained stable and symptom-free. He resumed his previously discontinued hobbies and driving, and maintained good overall functioning.

## Discussion

This case highlights a unique presentation of very late-onset schizophrenia-like psychosis (VLOSLP). The patient presented with profound apophenia, a tendency to perceive meaningful connections with unrelated or neutral stimuli. He described becoming preoccupied with perceived ‘algorithms’ in traffic flow, television signals, and buildings. These began to influence his behaviour and interpretation of events around him. This apophenia represents a failure of the brain's significance filter [13]. It can be a compensatory cognitive effort by the brain to resolve ambiguity generated by subcortical dopamine dysregulation [14].

In this specific case, MRI showed chronic white matter ischaemic changes and diffuse atrophy. These may represent predisposing factors that impaired processing and interpretation of environmental information, as VLOSLP has been associated with structural and functional brain changes [2]. A quick and sustained resolution of symptoms with olanzapine is consistent with evidence suggesting that late-onset schizophrenia-spectrum disorders may respond well to relatively low doses of antipsychotic medication, although careful monitoring is required due to increased sensitivity to side effects in older adults [3].

Limitations

Several limitations in this case report must be acknowledged. First, this is a single observational case study. The successful pharmacological management of apophenia with olanzapine cannot be broadly generalised to all patients with atypical VLOSLP. Secondly, the patient demonstrated sustained clinical remission extending over 14 months to January 2026. However, the presence of chronic white matter ischaemic changes on his baseline MRI complicates the exact aetiology. We lack advanced functional neuroimaging, such as Positron Emission Tomography (PET). Therefore, it is difficult to entirely uncouple his primary psychiatric presentation from the subtle effects of underlying microvascular disease.

## Conclusions

Very late-onset schizophrenia-like psychosis can present in unusual ways and may be difficult to distinguish from other medical and neurological conditions. This case shows that profound apophenic experiences, with the patient assigning special meaning to everyday environmental stimuli, may represent an atypical presentation of VLOSLP. Careful investigation to exclude secondary causes was essential before making the diagnosis. Management involved the use of olanzapine. Olanzapine was associated with a rapid and marked improvement, with apparent resolution of symptoms. Overall, this case highlights the importance of considering VLOSLP in older adults who present with new-onset psychotic symptoms and demonstrates that cautious, individualised treatment can lead to good clinical outcomes for the patient.
